# Diabetes distress and associated psychosocial factors in type 2 diabetes. A population-based cross-sectional study. The HUNT study, Norway

**DOI:** 10.1186/s13098-025-01631-w

**Published:** 2025-02-19

**Authors:** Hilde K.R. Riise, Anne Haugstvedt, Jannicke Igland, Marit Graue, Eirik Søfteland, Monica Hermann, Sofia Carlsson, Timothy C. Skinner, Bjørn Olav Åsvold, Marjolein M. Iversen

**Affiliations:** 1 Department of Health and Caring Sciences, Western Norway University of Applied Sciences, Bergen, Norway; 2 Department of Global Public Health and Primary Care, University of Bergen, Bergen, Norway; 3 Department of Clinical Medicine, University of Bergen, Bergen, Norway; 4 Haukeland University Hospital, Bergen, Norway; 5 Institute of Environmental Medicine, Karolinska Institutet, Stockholm, Sweden; 6 The Australian Centre for Behavioral Research in Diabetes, Melbourne, Australia; 7 HUNT Center for Molecular and Clinical Epidemiology, Department of Public Health and Nursing, NTNU, Norwegian University of Science and Technology, Trondheim, Norway; 8 Department of Endocrinology, St. Olavs Hospital, Trondheim University Hospital, Trondheim, Norway; 9 Department of Public Health and Nursing, HUNT Research Centre, NTNU, Norwegian University of Science and Technology, Levanger, Norway

**Keywords:** Diabetes mellitus, Diabetes Mellitus, type 2: distress, Anxiety, Depression

## Abstract

**Background and aim:**

The world-wide prevalence of diabetes distress varies, and studies are mainly undertaken in clinical settings. By using data from the Trøndelag Health (HUNT) study, we aimed to estimate diabetes distress prevalence, its determinants, and associations with anxiety and depression among adults with type 2 diabetes.

**Methods:**

This population-based cross-sectional study consists of individuals ≥ 20 years with type 2 diabetes participating in the HUNT4 survey (2017–2019). Diabetes-distress prevalence with 95% confidence interval (CI) was calculated based on the five item Problem Areas in Diabetes (PAID-5) questionnaire. PAID-5 sum scores were rescaled to a 0-100 scale by multiplying the sum score by five. Linear and logistic regression models were used to examine associations of demographic, lifestyle- and clinical factors, with diabetes distress.

**Results:**

In total, 1954 individuals completed the PAID-5 questionnaire, with a mean score of 15.2 (SD 18.3) and 11.9% (95% CI 10.6–13.4) reporting high diabetes distress (PAID-5 ≥ 40). Multivariable linear regression showed that diabetes distress was associated with a 0.2 (95% CI 0.2–0.3) lower score for each year older age, 7.6 (95% CI 5.4–9.7) higher score for current insulin use, and 9.3 (95% CI 5.3–13.2) higher score for a history of diabetes foot ulcers. High levels of anxiety and depression symptoms were associated with higher diabetes distress (Anxiety: B 16.0, 95% CI 13.6–18.4, Depression: B 13.3, 95% CI 10.7–16.0).

**Conclusions:**

Diabetes distress is common and strongly associated with younger age at diabetes onset, insulin use, foot ulcer, and anxiety and depression symptoms. Identifying and addressing diabetes distress in diabetes follow-up may facilitate improving health outcomes and prevent more serious mental health issues in individuals with T2D. Nevertheless, the findings should be further examined in longitudinal studies.

**Supplementary Information:**

The online version contains supplementary material available at 10.1186/s13098-025-01631-w.

## Introduction

Individuals with type 2 diabetes (T2D) may experience diabetes distress, commonly referred to as the emotional burden that arise from daily self-management of diabetes [1]. Adults with T2D perform complex self-management tasks to maintain adequate glycaemic and metabolic control with the intention of preventing short-term diabetes complications (e.g. hypoglycemia and diabetes keto acidosis) and preventing or delaying long-term diabetes complications (e.g. retinopathy, neuropathy, nephropathy, and cardiovascular disease) [2]. The efforts to self-manage the disease could be experienced as an emotional burden, and additionally feelings of guilt regarding blood glucose fluctuations and worry about developing complications, is not unusual [3]. 

A systematic review and metanalysis has reported a 36% worldwide prevalence of high diabetes distress in people with T2D [3], and that it is more prevalent in women, at younger age, among those with short duration of their disease, and in those with inadequate glycaemic control [4, 5]. Further, studies have shown that diabetes distress is associated with decreased glycaemic control, reduced adherence and self-care, increased risk of short- and long-term complications, and decreased quality of life [6]. Diabetes distress may interact severely with emotional well-being, and undetected and unaddressed diabetes distress may increase the risk of anxiety and depression. Studies on diabetes distress among people with T2D are predominantly undertaken in clinical settings (e.g., hospitals and clinical centres) Previous population-based studies such as the MILES study [7], the DAWN study [8], a German [9], and a US study [10], are as well limited with considerable selection bias. Thus, more knowledge from more representative population-based studies is needed. By using data from the fourth wave of the population based Trøndelag Health (HUNT) study in Norway, we aimed to (1) analyse the prevalence of diabetes distress among participants with T2D, and (2) examine associations of demographic, lifestyle- and clinical factors, including anxiety and depression, with diabetes distress.

## Methods

### The Trøndelag health study

In HUNT, an ongoing longitudinal population-based study in Norway, all inhabitants ≥ 20 years in the Nord-Trøndelag region have been invited to repeated surveys at 10-year intervals between 1984 and 2019 (HUNT1 1984/86, HUNT2 1995/97, HUNT3 2006/08, and HUNT4 2017/19). The characteristics of the HUNT population have been published previously [11]. HUNT includes extensive questionnaire data, clinical measurements, and biological samples, gathered by specially trained health personnel at field stations in each of the 23 municipalities in the region (https://www.ntnu.edu/hunt).

### Study population

In the current study we used data from HUNT4, conducted in 2017–2019. The study population in this community-based survey included all individuals ≥ 20 years who participated in HUNT4 and who also completed the diabetes-specific questionnaire. A total of 56,042 people (54.0% of those invited) participated in HUNT4. Of these, 3334 (5.9%) reported having diabetes and 2393 (71.8% of 3334) completed the diabetes-specific questionnaire. A total of 2042 participants had T2D, and of these, 1954 (95.7%) had valid data on the five item Problem Areas in Diabetes (PAID-5) questionnaire.

Type of diabetes was defined using glutamic acid decarboxylase antibodies (GADA) and self-reported age at diabetes diagnosis. In HUNT4 the unit of measure for GADA was international units per millimetre (IU/mL), while antibody index (a.i.) was used in the previous HUNT surveys. GADA was measured in serum samples and analysed by immunoprecipitation radioligand assay using translation labelled 3 H-GAD65 as a labelled reagent. T2D diabetes was defined as GADA < 5 IU/mL (< 0.08 a.i.) and age at diagnosis ≥ 30 years.

### Data material

#### Outcome variable

Diabetes distress was self-reported and measured using the PAID-5 questionnaire [12]. Each item on the PAID scale represents a unique area of diabetes-related psychosocial stress and higher scores indicate higher distress levels. In PAID-5 the participants are asked to rate the extent of each of the problem areas on a 5-point Likert scale (from 0 “not a problem” to 4 “serious problem”). A sum score is calculated by summing the score of the five questions, yielding a range of 0–20. A sum score ≥ 8 is defined as having high diabetes distress [12]. In the current study the PAID-5 sum scores were rescaled to a 0-100 scale by multiplying the sum score by five. This standardization allowed comparison with similar studies using PAID-20, a more extensive questionnaire on diabetes distress. A cut-off ≥ 40 (corresponding to ≥ 8 without rescaling) was used to define diabetes distress. Both PAID-20 and PAID-5 have shown good reliability and validity among adults with type 1 and T2D [13, 14]. In addition, the PAID-5 total score has been found to correlate significantly with the PAID-20 total score [12]. 

#### Exposure variables

##### Questionnaire data

The following information on sociodemographic status was collected from the main questionnaire: education (primary school/high school/vocational school vs. college/university), living alone (marital status), and occupational status (currently employed yes/no). The national identification number provided information on age and sex. Also, information on the following health-related variables was available from the main questionnaire: smoking (current daily smoking), use of antihypertensive drugs, physical activity (ones or less a week, more than once a week), and previous disease (stroke, myocardial infarction, angina pectoris). Lastly, we retrieved information on the following diabetes-related variables from the diabetic-specific questionnaire: diabetes duration, use of glucose lowering medications other than insulin, current use of insulin, reported diabetes-related eye problems, and history of foot ulcer.

Symptoms of anxiety and depression were measured using the Hospital depression and anxiety scale (HADS-D and HADS-A). The questionnaire is comprised of seven questions for anxiety and seven questions for depression [15]. Each question is scored from 0 to 3; thus, the maximum score is 21 on each of the subscales. Higher scores indicate higher levels of symptom load. A subscale score of ≥ 8 denotes mild to serious symptom load indicating possible anxiety or depression disorder. The HADS questionnaire has been validated in many languages and countries [16]. 

##### Clinical variables

The following data from clinical examinations were retrieved: body mass index (BMI), systolic blood pressure (> 140 mm Hg (> 18.7 kPa)), and HbA1c. Height and weight were measured and BMI (weight in kilograms divided by the squared value of height in meters) was calculated. Blood pressure was measured three times, and the mean value of the second and third measurements were used. Non-fasting blood samples were collected and handled according to appropriate standards. HbA1c was used both as a continuous and as a categorial variable (≤ 7.0% (≤ 53 mmol/mol), 7.1–7.5% (54–58 mmol/mol), 7.6-8.0% (59–64 mmol/mol), 8.1–8.9% (65–74 mmol/mol) and ≥ 9.0% (≥ 75 mmol/mol)).

### Statistical analyses

Analyses were performed using Stata software (StataCorp, 2019, Stata Statistical Software: Release 17, College Station, TX: StataCorp LLC). Descriptive statistics were reported as frequencies and percentages for categorical variables and means and standard deviations (SD) for continuous variables.

To analyse the prevalence of high diabetes distress, we used the PAID-5 sum score as a categorical variable with a cut-off score ≥ 40 indicating high distress. Diabetes distress prevalence with 95% confidence intervals (CI) was estimated for the total sample and stratified by sociodemographic, lifestyle and clinical factors. CI was estimated using the logit-method [17]. We also calculated the proportion of participants scoring somewhat serious (score 3) or serious (score 4) on each item to identify the most prevalent aspects of distress.

To examine associations of demographic, lifestyle and clinical factors with the categorical diabetes distress variable (PAID-5 ≥ 40) we used logistic regression. Results are reported as odds ratios with 95% CI based on standard asymptotic methods. For the continuous PAID-5 sum score we used linear regression. We report the difference (with 95% CI based on the t-distribution and standard asymptotic methods from ordinary least squares estimation) in the PAID-5 score per one-unit higher value of each exposure variable. We examined normal distribution and homoscedasticity, and no clear deviation from the model assumptions were found. Adjustment variables included age, sex, education, BMI, exercise, diabetes duration, HbA1c and current use of insulin. We report two levels of adjustment. One model adjusted for age and sex, another additionally adjusted for relevant confounders that were defined separately for each exposure variable based on clinical knowledge. A Venn diagram with overlapping circles was created to show commonalities and differences in reported diabetes distress, and anxiety and depression symptoms.

### Ethics

This study was approved by the Regional Committees for Medical Research and Health Research Ethics in Norway (74975) and the Norwegian Centre for Research Data (150393). All participants gave informed consent.

## Results

Table 1 shows the characteristics of the study population, in total and stratified by age categories (< 50, 50–64 and ≥ 65 years). The average age of the participants was 67.3 years, (range 31.3–94.7). A total of 57.2% of the participants were men, and 30% had higher education. Mean duration of T2D was 12.1 years (SD, 9.4 years), and mean HbA1c was 51 mmol/mol (6.8%; SD, 1.0 years)). A total of 22.7% reported to be using insulin with or without other glucose- lowering medications, while 76.2% did not report insulin use.

**Table 1:** Sociodemographic status and health characteristics of the HUNT4 population with known type 2 diabetes

	Total study population (PAID responders)		Age < 50	Age 50–64	Age ≥ 65
**Predictors**					
**Overall**	1954 (100)		140 (7.2)	572 (29.3)	1242 (63.6)
**Socio-demographics status**					
Women, n (%)	837 (42.8)		81 (57.9)	234 (40.9)	522 (42.1)
Living alone, n (%)	713 (36.5)		76 (54.3)	214 (37.4)	423 (34.1)
College/university, n (%)	525 (27.0)		56 (40.0)	180 (31.5)	289 (23.5)
Currently employed, n (%)	586 (30.0)		114 (81.4)	393 (68.8)	79 (6.4)
**Lifestyle factors**					
Body mass index					
≤ 25, n (%)	407 (21.0)		13 (9.3)	98 (17.1)	296 (24.2)
26–30, n (%)	811 (41.9)		38 (27.1)	236 (41.3)	537 (43.9)
31–34, n (%)	434 (22.4)		44 (31.4)	140 (24.5)	250 (20.3)
≥ 35, n (%)	285 (14.7)		45 (32.1)	98 (17.1)	142 (11.6)
Current daily smoking, n (%)	152 (7.9)		11 (7.9)	68 (11.9)	73 (5.9)
Exercise					
Once or less a week, n (%)	389 (20.4)		38 (27.3)	115 (20.3)	236 (19.6)
More than once a week, n (%)	1521 (79.6)		101 (72.7)	451 (79.7)	969 (80.4)
**Clinical characteristics**					
Systolic blood pressure > 140 mm Hg (18.7 kPa), n (%)	735 (37.6)		22 (15.7)	176 (30.8)	537 (43.2)
Using antihypertensive drugs, n (%)	1299 (72.7)		42 (37.2)	339 (64.2)	918 (80.0)
Duration of diabetes					
< 1 years, n (%)	63 (3.6)		11 (8.9)	18 (3.3)	34 (3.1)
1–4 years, n (%)	369 (20.8)		46 (37.1)	157 (29.1)	166 (15.0)
5–9 years, n (%)	353 (19.9)		32 (25.8)	125 (23.2)	196 (17.7)
≥ 10 years, n (%)	989 (55.8)		35 (28.2)	240 (44.4)	714 (64.3)
**Glycemic control**					
HbA1c %, mean (SD)	6.8 (1.0)		6.6 (1.3)	6.8 (1.0)	6.8 (1.0)
HbA1c mmol/mol, mean (SD)	51.1 (11.0)		48.4 (13.8)	51.3 (11.1)	51.3 (10.6)
Using other glucose-lowering medications, n (%)	1478 (76.2)		84 (60.0)	432 (75.8)	962 (78.3)
Using insulin now, n (%)	421 (22.7)		23 (16.8)	98 (17.9)	300 (25.6)
**Microvascular complications**					
Diabetes eye- problems, n (%)	150 (7.8)		9 (6.4)	33 (5.8)	108 (8.9)
Diabetes foot ulcers, n (%)	97 (5.0)		10 (7.1)	27 (4.8)	60 (4.9)
**Microvascular complications**					
Acute myocardial infarction, n (%)	242 (13.4)		2 (1.5)	53 (9.9)	187 (16.6)
Cerebral stroke, n (%)	150 (8.5)		3 (2.1)	23 (4.3)	124 (11.2)
Angina pectoris, n (%)	172 (9.7)		2 (1.5)	30 (5.6)	140 (12.8)

SD indicates standard deviation

**Fig. 1:**
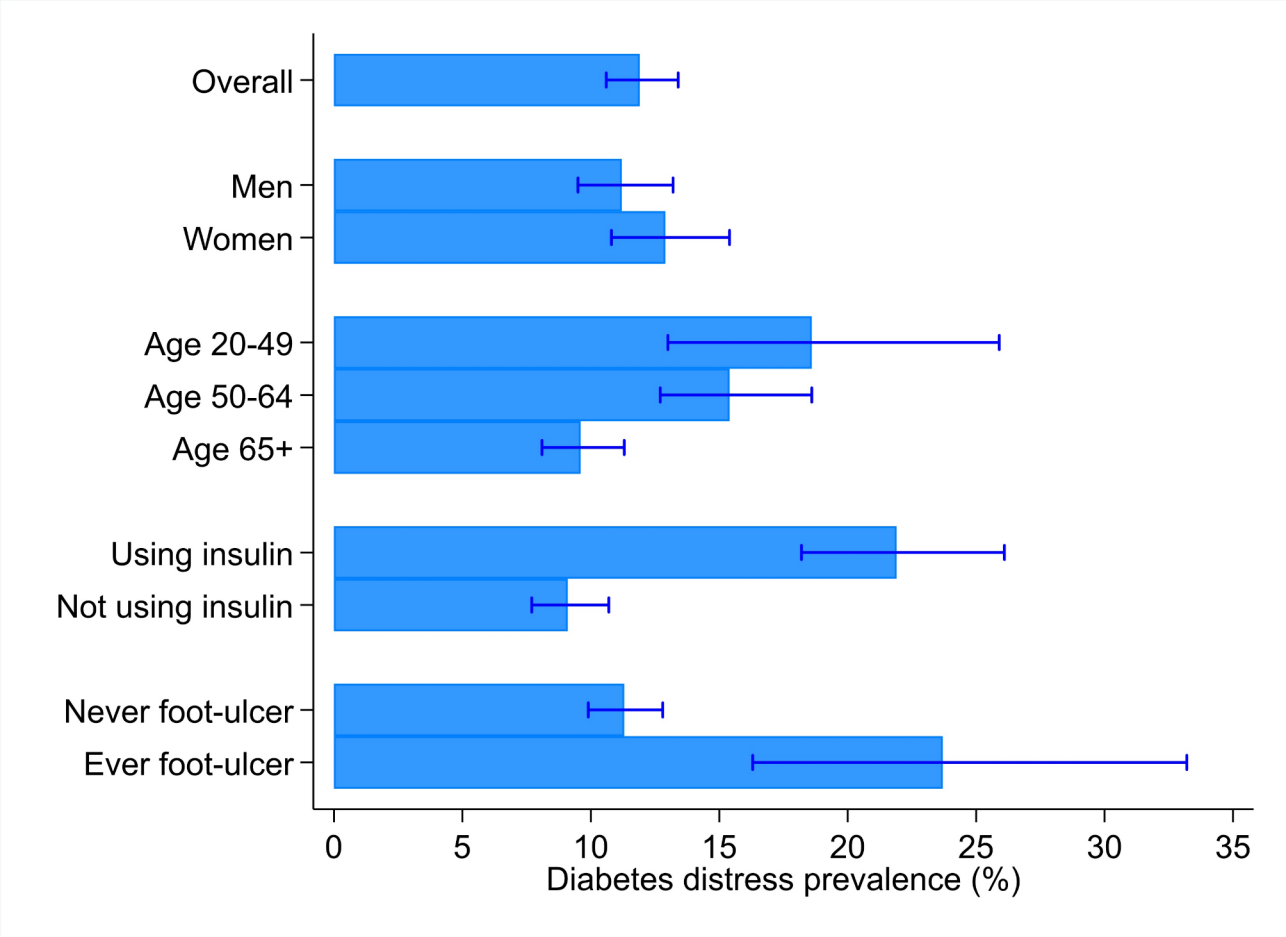
High diabetes distress point prevalence (%) across key sociodemographic- and clinical factors. Error bars are 95% confidence intervals

The overall prevalence of high diabetes distress was 11.9% (95% CI 10.6–13.4) (Table 2; Fig. 1). The proportion of participants with high diabetes distress was higher in those with young age at participation (< 50) and young age at diabetes onset (< 50). High diabetes distress was also more prevalent in participants using insulin, and in those reporting a history of footulcers.

**Table 2:** High diabetes distress point prevalence (%) across sociodemographic, lifestyle- and clinical factors among HUNT4 participants

	*N* (PAID responders)	Point prevalence high diabetes distress (Rescaled PAID-5 sum score ≥ 40) 95% CI)	Model 1* Odds ratio (95% CI)	Model 2≠ Odds ratio (95% CI)
**Overall**	1954	11.9 (10.6, 13.4)		
**Socio-demographics**				
Sex				
Men	1117	11.2 (9.5, 13.2)	Ref.	
Women	837	12.9 (10.8, 15.4)	1.1 (0.9, 1.5)	
Age at participation				
20–49 yrs.	140	18.6 (13.0, 25.9)	Ref.	
50–64 yrs.	1354	15.4 (12.7, 18.6)	0.8 (0.5, 1.3)	
≥65 yrs.	460	9.6 (8.1, 11.3)	0.5 (0.3, 0.8)	
Age at diabetes diagnosis				
20–49 yrs.	572	17.5 (14.6, 20.9)	Ref.	
50–64 yrs.	912	9.9 (8.1, 12.0)	0.5 (0.4, 0.7)	
≥65 yrs.	352	8.4 (5.9, 11.9)	0.4 (0.3, 0.7)	
Marital status				
Not living alone	1239	11.1 (9.5, 13.0)	Ref.	
Living alone	713	13.3 (11.0, 16.0)	0.9 (0.7, 1.1)	
Educational level				
No college/university	1418	11.0 (9.5, 12.7)	Ref.	
College/university	525	14.1 (11.4, 17.3)	1.2 (0.9, 1.7)	
**Lifestyle factors**				
BMI				
≤25	407	9.3 (6.9, 12.6)	Ref.	Ref.
26–30	811	11.2 (10.7, 17.2)	1.2 (0.8, 1.8)	1.1 (0.7, 1.6)
>30	719	13.6 (10.7, 17.2)	1.4 (0.9, 2.0)	1.2 (0.8, 1.9)
Smoking				
Not current smoker	1785	11.8 (10.3-13-3)	Ref.	Ref.
Current smoker	152	18.8 (9.2, 20.3)	1.1 (0.7, 1.8)	1.1 (0.7, 1.8)
Exercise				
≤ 1 h a week	389	10.0 (7.4, 13.4)	Ref.	Ref.
>1 h a week	1521	12.4 (10.8, 14.1)	1.3 (0.9, 1.8)	1.3 (0.9, 1.9)
**Clinical factors**				
Duration of diabetes				
<1 year	63	20.6 (12.4, 32.4)	Ref.	Ref.
≥ 1 year	1711	11.7 (10.2, 13.3)	0.8 (0.6, 1.3)	0.6 (0.3, 1.1)
Hypertension (systolic blood pressure > 140)				
No	1219	13.1 (11.3, 15.1)	Ref.	Ref.
Yes	735	9.9 (8.0-12.3)	0.8 (0.6, 1.1)	0.9 (0.6, 1.2)
**Glycemic control**				
HbA1c				
On target (≤ 53 mmol/mol (≤ 7.0%))	1303	10.4 (8.8, 12.1)	Ref.	Ref.
not on target (> 53 mmol/mol (> 7.0%))	621	15.3 (12.7, 18.4)	1.6 (1.2, 2.2)	1.3 (0.9, 1.8)
Using insulin now	30			
Yes	421	21.9 (18.2, 26.1)	3.2 (2.3, 4.3)	2.9 (2.1, 4.1)
No	1438	9.1 (7.7, 10.7)	Ref.	Ref.
**Microvascular complications**				
Diabetes eye- problems				
No	1444	10.8 (9.3, 12.5)	Ref.	Ref.
Yes	150	17.3 (12.1, 24.2)	1.9 (1.2, 3.8)	1.3 (0.8, 2.2)
Diabetes foot ulcers				
No	1831	11.3 (9.9, 12.8)	Ref.	Ref.
Yes	97	23.7 (16.3, 33.2)	2.5 (1.5, 4.2)	2.6 (1.5, 4.4)

CI indicates confidence interval

PAID-5 sum scores were rescaled to a range between 0-100 by multiplying the sum score by five

* Adjustment variables in model 1: age and sex

≠ adjustment variables in model 2. Separate models for each variable of interest with adjustment for relevant confounders. Model for smoking/duration of diabetes adjusted for age, sex, and education; Model for education adjusted for age and sex; Model for BMI adjusted for age, sex, education, exercise, HbA1c, duration of diabetes, and current use of insulin; Model for HbA1c adjusted for age, sex, education, duration of diabetes and use of insulin; Model for hypertension/use of insulin adjusted for age, sex, education, HbA1c, and duration of diabetes; Model for history of diabetes foot ulcer/diabetes eye problems adjusted for age, sex, education, HbA1c, duration of diabetes, and current use of insulin

The PAID-5 items most often endorsed as a somewhat serious (score 3) or serious (score 4) problem were item 3: “worrying about the future and the possibility of serious complications” and item 5: “coping with complications of diabetes” (Supplementary Tables 1 and 2). Of the participants reporting low levels of diabetes distress (PAID-5 score ≤ 40) approximately 40% reported to have minor and/or moderate problems with the same two items (Supplementary Table 2).

Multivariable linear regression (Table 3) confirmed that diabetes distress was positively associated with younger age. Compared to older participants (≥ 65 years), mean PAID-5 sum score was 4.4 (95% CI 1.2–7.6) points higher among participants aged 20–49 years, and 4.3 (95% CI 2.5–6.1) points higher among participants aged 50–64 years. Diabetes distress was also higher among insulin users (B 7.6, 95% CI 5.4–9.7), and among people with a history of diabetes foot ulcers (B 9.3, 95% CI 5.3–13.2). No association was found between macrovascular complications (acute myocardial infarction, cerebral stroke, and angina pectoris) and diabetes distress (results not shown in table). Analysis of associations of anxiety and depression symptoms with diabetes distress are shown in Table 4. We found strong associations between anxiety symptoms and diabetes distress (B 16.0, 95% CI 13.6,18.4) and between depression symptoms and diabetes distress (B 13.3, 95% CI 10.7–16.0).

**Table 3:** Associations of demographic variables, lifestyle factors, clinical factors and glycemic control with continuous PAID-5 scores

	Diabetes distress (PAID-5 sum score ≥ 40)						
	Unadjusted			Model 1*		Model 2≠	
	Mean	B	95% CI	B	95% CI	B	95% CI
**Socio-demographics**							
Sex							
Men	14.8	Ref.		Ref.			
Women	15.7	0.9	-0.7, 2.6	0.8	-0.8, 2.4		
Age-groups							
20–49 yrs.	18.0	4.4	1.2, 7.6	4.3	1.1, 7.5		
50–64 yrs.	17.9	4.3	2.5, 6.1	4.3	2.5, 6.1		
≥65 years	13.6	Ref.		Ref.			
Age at diabetes diagnosis							
20–49 yrs.		7.0	4.5, 9.4	6.9	4.5, 9.4		
50–64 yrs.		2.2	-0.1, 4.5	2.3	-0.0, 4.5		
≥65 yrs.		Ref.		Ref.			
Marital status							
Not living alone	14.7	Ref.		Ref.			
Living alone	16.0	1.3	-0.4, 3.0	1.0	-0.7, 2.6		
Educational level							
No college/university	14.5	Ref.		Ref.			
College/university	16.7	2.1	0.3, 4.0	1.6	-0.2, 3.4		
**Lifestyle factors**							
BMI							
≤25	13.7	Ref.		Ref.		Ref.	
26–30	15.0	1.3	-0.9, 3.5	0.9	-1.3, 3.1	0.1	-2.2, 2.5
>30	16.1	2.4	0.2, 4.6	1.3	-0.9, 3.6	0.4	-2.0, 2.9
Smoking							
Not current smoker	15.1	Ref.		Ref.		Ref.	
Current smoker	16.2	1.0	-2.0, 4.1	0.3	-0.2, 3.3	0.6	-2.5, 3.6
Exercise							
≤ 1 h a week	13.2	Ref.		Ref.		Ref.	
>1 a week	15.7	2.5	0.5, 4.6	2.5	0.5, 4.6	2.6	0.6, 4.6
**Clinical factors**							
Duration of diabetes							
<1 year	19.6	Ref.					
≥1 year	15.2	-4	-8.9, -0.3	-3.5	-8.1, 1.1	-3.5	-8.1, 1.0
Hypertension (systolic blood pressure > 140)							
No	15.7	Ref.		Ref.		Ref.	
Yes	14.3	-1.3	-3.0, 0.3	-0.6	-2.3, 1.1	-0.5	-2.3, 1.3
**Glycemic control**							
HbA1c							
On target (≤ 53 mmol/mol (≤ 7.0%))	13.4	Ref.		Ref.		Ref.	
not on target (> 53 mmol/mol (> 7.0%))	16.1	2.7	1.0, 4.4	3.2	1.5, 4.9	1.6	-0.2, 3.5
Current use of insulin							
No	13.5	Ref.		Ref.		Ref.	
Yes	21.4	7.9	6.0, 9.9	8.6	6.6, 10.6	7.6	5.4, 9.7
**Microvascular complications**							
History of diabetes foot ulcer							
No	14.6	Ref.		Ref.		Ref.	
Yes	24.5	8.6	1.1, 16.3	10.1	6.4, 13.8	9.3	5.3, 13.2
Diabetes eye problems							
No	14.5	Ref.		Ref.		Ref.	
Yes	19.5	4.9	1.9, 8.0	5.7	2.6, 8.8	2.6	-0.7, 5.8

B = regression coefficient, interpreted as mean difference in continuous PAID-5 score. CI = Confidence Interval

PAID-5 sum score rescaled to a range between 0-100

* Adjustment variables in model 1: age and sex

≠ adjustment variables in model 2. Separate models for each variable of interest with adjustment for relevant confounders. Model for smoking/duration of diabetes adjusted for age, sex, and education; Model for education adjusted for age and sex; Model for BMI adjusted for age, sex, education, exercise, HbA1c, duration of diabetes, and current use of insulin; Model for HbA1c adjusted for age, sex, education, duration of diabetes and use of insulin; Model for hypertension/use of insulin adjusted for age, sex, education, HbA1c, and duration of diabetes; Model for history of diabetes foot ulcer/diabetes eye problems adjusted for age, sex, education, HbA1c, duration of diabetes, and current use of insulin

**Table 4:** Associations of high anxiety and depression symptoms with diabetes distress in type 2 diabetes

	Diabetes distress (PAID-5, 0-100 scale)							
	Unadjusted					Adjusted^*^		
	n	PAID mean score	B	95% CI	*P*-value	B	95% CI	*P*-value
**Self-reported psychological health**								
HADS anxiety, continuous			2.1	1.9, 2.4	< 0.001	2.2	2.0, 2.5	< 0.001
HADS anxiety								
0–7 (normal)	1485	12.5	Ref.			Ref.		
8–21 (mild- serious symptoms load)	292	28.3	15.9	13.7, 18.0	< 0.001	16.0	13.6, 18.4	< 0.001
HADS depression, continuous			1.8	1.5, 2.1	< 0.001	1.9	1.7, 2.2	< 0.001
HADS depression								
0–7 (normal)	1576	13.5	Ref.			Ref.		
8–21 (mild-serious symptom load)	219	27.0	13.4	11.0, 15.9	< 0.001	13.6	10.9, 16.3	< 0.001

* Adjusted for age, sex, education, diabetes duration, HbA1c, current use of insulin

**Fig. 2:**
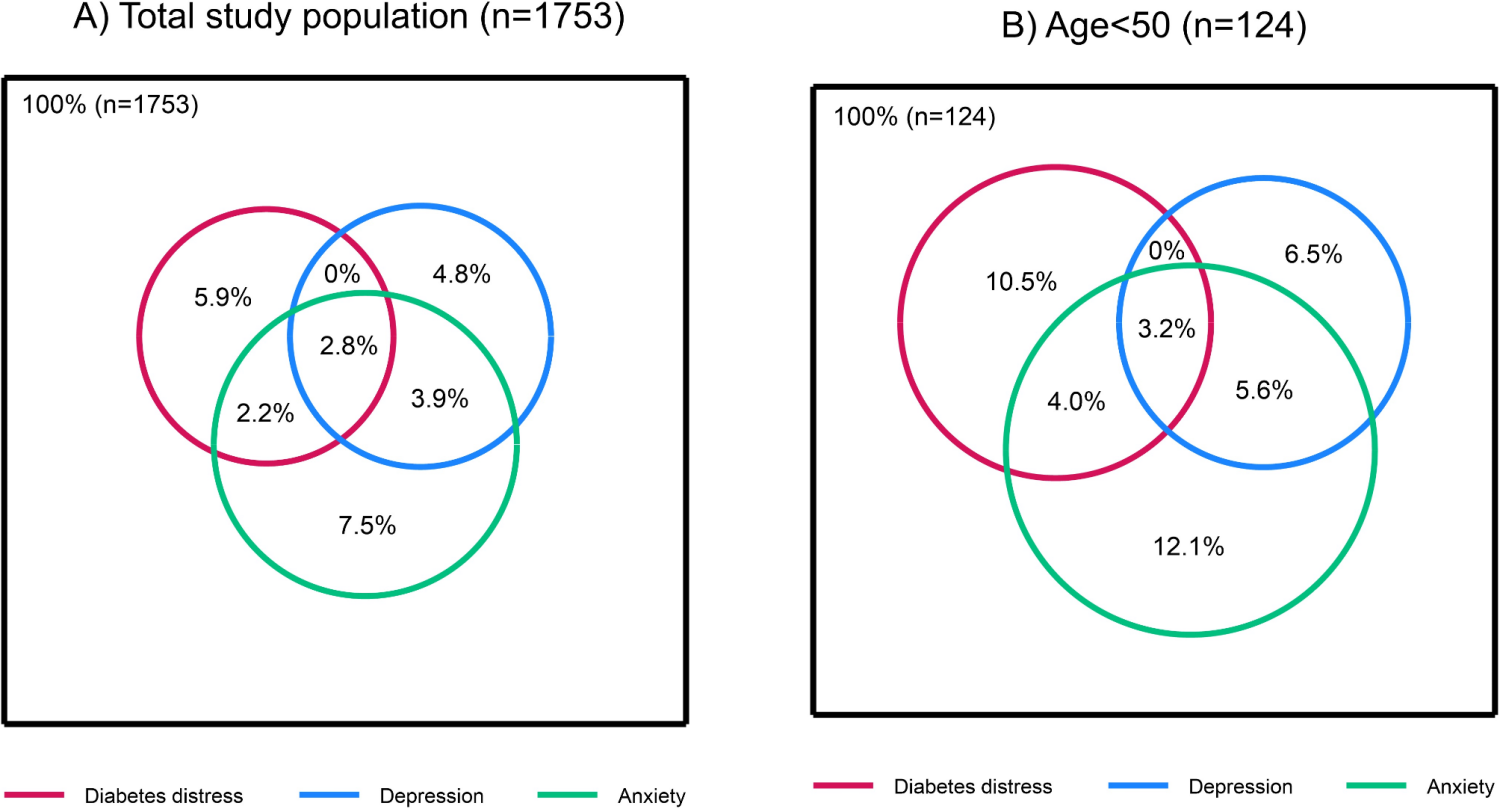
Percentage of participants with high diabetes distress, and/or high anxiety symptoms, and/or high depression symptoms. (**A**) the total study population, and (**B**) those < 50 years of age. The study population used to construct the diagrams includes individuals with non-missing values on all three variables. The squared area reflects the size of the study population used to construct the diagram. The area of the circles in relation to the area of the square reflects the proportion of the study population with distress, anxiety or depression.

Among the participants, 16.4% (12.2% of men and 22.0% of women) scored above cut-off for mild to serious level of anxiety symptoms (HADS-A ≥ 8) (Supplementary Table 3). Regarding symptoms of depression, 12.2% (12.6% of men and 11.6% of women) scored above cut-off for mild to serious level of depression symptoms (HADS-D ≥ 8). In Fig. 2 we present the distribution of participants with high diabetes distress (PAID > 5 ≥ 40) and/or high levels of anxiety symptoms (HADS-A score ≥ 8) and/or high levels of depression symptoms (HADS-d score ≥ 8), in the total population and among those < 50 years of age. More people reported simultaneous high diabetes distress and anxiety symptoms than simultaneous high diabetes distress and depression symptoms.

## Discussion

This population-based cross-sectional study assessed the prevalence of diabetes distress among people with T2D in Norway. Of 1954 participants with T2D, only 11.9% reported high diabetes distress. The two items on worries for future complications and coping with complications contributed the most to higher distress scores. We identified significant associations between diabetes distress and young age at diabetes onset, insulin use, and a history of diabetes foot ulcer. However, we did not identify any association between diabetes distress and decreased glycaemic control (HbA1c) when adjusted for insulin use. We found strong associations between diabetes distress and depression symptoms, and even stronger associations with anxiety symptoms. More people reported concurrent diabetes distress and anxiety symptoms than concurrent diabetes distress and depression symptoms. Our findings highlight the importance of identifying diabetes distress and its impacts on clinical factors included anxiety and depression among individuals with T2D.

The prevalence of high diabetes distress in the current study is substantially lower compared to estimates from previous studies [3], which mainly are conducted in clinical settings. Even though a few population-based studies have been identified [7–10], these studies have significant limitations with regards to selection bias and low participation rates, which has a considerable impact on the generalizability of the results. The low prevalence rate identified in this study may indicate that the general population of people with T2D is coping better with their diabetes than typically seen in participants in certain samples recruited to specific intervention and hospital-based studies. The different prevalence of diabetes distress observed across various populations underlines the need to take methodological factors into account when interpreting study results. In addition, advances in clinical and preventive care may play a role in reducing high diabetes distress among people with T2D. Accordingly, a previous publication has indicated improved mental health in the general Norwegian population from 1995 to 2019 [18], which also is similar to trends in other populations [19]. 

The associations identified between high diabetes distress and younger age, current use of insulin, and a history of diabetes foot ulcer are in line with previous studies [5, 6]. T2D with early onset is associated with more aggressive disease, and further higher levels of diabetes distress [20], supporting the need for clinical vigilance and age-appropriate psychosocial support [21]. Also, people with T2D are likely to experience shame or guilt associated with their disease due to information from the media suggesting that lifestyle choices and eating habits are the main reasons for the T2D diagnosis. We also found that use of insulin was strongly associated with diabetes distress. Insulin treatment adds challenges to self-care, such as the need to self-inject and determine insulin doses in relation to meals and activity. In addition, starting insulin treatment is often a sign of disease progression that may further increase the fears of diabetes complications. Lastly, the association between a history of diabetes foot ulcers and increased diabetes distress may also be caused by a feeling of disease progression, increased health care needs, risk of amputation, and feelings of fear and loss of mobility [22]. Hence, it is essential to identify and address distress in people with T2D early.

In contrast to previous studies [23], we did not find an association between decreased glycaemic control (HbA1c) and high diabetes distress after adjusting for use of insulin. It seems that people with T2D struggle more with the insulin treatment itself than worrying about glycaemic control and its potential consequences. Having to inject insulin every day is often perceived as more invasive than oral treatment. Those who in addition need to assess insulin doses themselves, may also experience low confidence in their own abilities to carry out the treatment. Thus, clinicians should be aware that people treated with insulin may experience higher diabetes distress.

In this study, the PAID-5 items most strongly endorsed as problematic were concerns about serious complications and coping with complications of diabetes, whereas feeling scared or feeling depressed when thinking about living with diabetes were not as common sources of distress. These findings are consistent with studies from countries with comparable living conditions and health care services [24]. A constant worry about complications and a constant effort to manage the treatment may result in a feeling of burnout and highlights the importance of addressing diabetes distress in clinical consultations. Furthermore, previous research has shown that people with T2D exaggerate risk estimations regarding long-term complications [25], leading to unnecessary levels of distress. Knowledge of one’s own risk for complications can help to reduce distress.

The 12.3% prevalence of HADS-D ≥ 8 indicating possible depression is lower than reported in previous studies [26]. However, the identified 16.5% prevalence of HADS-A ≥ 8 indicating high levels of anxiety symptoms is more in line with previous studies [27]. Nevertheless, the diabetes population in the current study seemed to have a higher anxiety and depression symptom load than the general population [28, 29]. It has been shown that people with diabetes have an average of 30% higher risk of developing depression than those without diabetes [30], and that anxiety disorders and anxiety symptoms are present in respectively 14% and 40% of people with diabetes [31]. While the prevalence of possible depression was equal between men and women in the current study, women reported more anxiety than men (22.1% vs. 12.3%). The predominance of anxiety symptoms in women may reflect the well-documented overall sex pattern for anxiety disorders reported in previous studies [32]. We further observed that more people reported symptoms of anxiety, with or without coexisting diabetes distress, than symptoms of depression. An anxious coping style, such as avoidance and denial, can reduce adherence to self-care and negatively impact glycaemic control [33], with a risk of further exacerbating diabetes distress. Moreover, uncertainties and worries related to the risk of diabetes complications and the ongoing demands of disease treatment cause an emotional burden that can contribute to anxiety. Individuals with diabetes may experience lack of control over own health, which is a well-documented trigger for anxiety, particularly in those managing chronic conditions [34]. While depression remains a considerable concern for individuals with diabetes, the dynamic and unpredictable nature of diabetes may make anxiety a more immediate and dominant emotional response.

The management of a chronic condition such as T2D represents a substantial source of psychological distress for many individuals. Further, diabetes distress is shown to be a significant barrier for achieving satisfactory glycemic control [35]. Therefore, elevated levels of diabetes distress should be recognized as clinically important, and efforts should be made to increase the awareness of distress and other mental health issues in individuals with T2D. We recommend regularly assessment of diabetes distress to avoid untreated health issues in individuals with T2D, and also to mitigate the potential exacerbation of diabetes distress. Special attention should be paid to those treated with insulin, the youngest ones, those with pervious diabetes foot ulcer, and those with a history of mental anxiety and/or depression.

### Strengths and limitations

The main strength of the current study is the population-based study design and the high participation rate, which increases representativeness. The large study sample adds weight to previous diabetes distress studies with smaller and more selected populations. However, as with all large-scale-epidemiological studies, this study also has some limitations. Diabetes was self-reported but has shown high validity in HUNT with a specificity of 99.5% and a sensitivity of 81% or more [36], and should therefore only result in a small number of misclassifications. Also, the information on micro- and macrovascular complications was self-reported. In addition, the participants in HUNT are recruited from Trøndelag county, a specific geographical area in Norway with few large cities. The population is relatively homogeneous with less than 3% non-Caucasians, and it is generally stable [36]. No differences in demographic status and clinical variables were found between the total diabetes population in HUNT4 and the PAID responders (numbers not shown). Finally, the cross-sectional study design prohibits causal inferences from being made.

## Conclusions

In this community-based survey we identified a substantially lower prevalence of high diabetes distress than previously reported in specific intervention and hospital-based studies. Nonetheless, the observed associations between diabetes distress and younger age at diabetes onset, insulin use, foot ulcer, as well as anxiety and depression symptoms highlight the necessity of identifying and addressing diabetes distress in ongoing diabetes follow-up. This will facilitate improvements in health outcomes and help prevent more serious mental health issues among individuals with T2D. Nevertheless, the findings should be further examined in longitudinal studies.

## Electronic supplementary material

Below is the link to the electronic supplementary material.

Supplementary Material 1

Supplementary Material 2

## Data Availability

The data that support the findings of this study are available from HUNT, but restrictions apply to the availability of these data, which were used under license for the current study and therefore are not publicly available. Anonymous data are however available from the authors upon reasonable request and with permission from HUNT and the Regional Committees for Medical and Health Research Ethics in Norway.

## Abbreviations

ai: antibody index
BMI: Body mass index
CI: Confidence interval
GADA: glycemic acid decarboxylase antibodies
HADS-D and HADS-A: Hospital depression and anxiety scale
HUNT study: Trøndelag Health study
IU/mL: international units per millimetre
PAID-5: The five item Problem Areas in Diabetes questionnaire
SD: Standard deviation
T2D: Type 2 diabetes (T2D)
