# Supplementary material for: Diabetes distress and associated psychosocial factors in type 2 diabetes. A population-based cross-sectional study. The HUNT study, Norway
Source: Diabetol Metab Syndr. 2025 Feb 19;17:62. doi: 10.1186/s13098-025-01631-w (PMC11837721; doi:10.1186/s13098-025-01631-w)
Supplement: Supplementary file 1 — Supplementary Material 1 [file 13098_2025_1631_MOESM1_ESM.docx]

*Supplementary material*

*Supplementary Table 1: Frequency of PAID-5 item scores among participants with known diabetes type 2 in HUNT 4* (N=1954)

|  | 0. Not a problem | 1. Minor problem | 2. Moderate problem | 3. Somewhat serious problem | 4. Serious problem | Missing | Score 3-4 |
| --- | --- | --- | --- | --- | --- | --- | --- |
| **Question PAID-5** | n (%) | n (%) | n (%) | n (%) | n (%) | n (%) | n (%) |
| 1. Feeling scared when you think about living with diabetes | 1188 (58.2) | 508 (24.9) | 223 (10.9) | 44 (2.2) | 11 (0.5) | 68 (3.33) | 55 (2.7) |
| 2. Feeling depressed when you think about living with diabetes | 1327 (65.0) | 448 (21.9) | 138 (6.8) | 30 (1.5) | 11 (0.5) | 88 (4.31) | 41 (2.0) |
| 3. Worrying about the future and the possibility of serious complications | 1005 (49.2) | 550 (26.9) | 293 (14.4) | 73 (3.6) | 29 (1.4) | 92 (4.51) | 102 (5.0) |
| 4. Feeling that diabetes is taking up too much of your mental and physical energy every day | 1270 (62.2) | 445 (21.8) | 171 (8.4) | 50 (2.5) | 15 (0.7) | 91 (4.46) | 65 (3.2) |
| 5. Coping with complications of diabetes | 967 (47.4) | 567 (27.8) | 281 (13.8) | 78 (3.8) | 24 (1.2) | 125 (6.12) | 102 (5.0) |

Supplementary Table 2: *Frequency of PAID-5 item scores among participants with known diabetes type 2 in HUNT 4* with PAID-5 sum score <40 (indicating no clinically significant diabetes distress) (N=1954)

|  | 0. Not a problem | 1. Minor problem | 2. Moderate problem | 3. Somewhat serious problem | 4. Serious problem | Missing |
| --- | --- | --- | --- | --- | --- | --- |
| **Question PAID-5** | n (%) | n (%) | n (%) | n (%) | n (%) | n (%) |
| 1. Feeling scared when you think about living with diabetes | 1174 (68.2) | 468 (27.2) | 76 (4.4) | 2 (0.2) | - | 1 (0.1) |
| 2. Feeling depressed when you think about living with diabetes | 1316 (76.5) | 384 (22.3) | 20 (1.2) | - | - | 1 (0.1) |
| 3. Worrying about the future and the possibility of serious complications | 1003 (58.3) | 532 (30.9) | 168 (9.8) | 14 (0.8) | - | 4 (0.2) |
| 4. Feeling that diabetes is taking up too much of your mental and physical energy every day | 1261 (73.3) | 388 (22.6) | 62 (3.6) | 5 (0.3) | 1 (0.1) | 4 (0.2) |
| 5. Coping with complications of diabetes | 964 (56.0) | 540 (31.4) | 158 (9.2) | 18 (1.1) | 4 (0.2) | 37 (2.2) |

Supplementary Table 3: Point prevalence (%) of likely anxiety (HADS-A) and depression (HADS-D) across sex and age among PAID responders in HUNT4 (N=1954)

|  | **Point prevalence anxiety (HADS-D sum score ≥8) (95% CI)** | **Point prevalence depression (HADS-A sum score ≥8) (95% CI)** |
| --- | --- | --- |
| **Overall** | 16.4 (14.8-18.2) | 12.2 (10.8-13.8) |
| **Sex** |  |  |
| Men | 12.2 (10.4-14.4) | 12.6 (10.7-14.8) |
| Women | 22.0 (19.2-25.1) | 11.6 (9.5-14.1) |
| **Age groups** |  |  |
| 20-49 yrs. | 25.4 (18.6-33.7) | 15.2 (9.9-22.6) |
| 50-64 yrs. | 21.5 (18.2-25.2) | 13.2 (10.6-16.4) |
| ≥65 yrs. | 13.1 (11.3-15.2) | 11.4 (9.7-13.4) |
| **Age at diabetes diagnosis** |  |  |
| 20-49 yrs. | 22.4 (19.0-23.3) | 15.1 (12.2-18.6) |
| 50-64 yrs. | 14.7 (12.4-17.3) | 11.9 (9.8-14.2) |
| ≥65 yrs. | 9.6 (12.4-17.3) | 9.6 (6.8-13.4) |
| **Marital status** |  |  |
| Not living alone | 14.8 (12.8-17.0) | 11.5 (9.8-13.5) |
| Living alone | 19.4 (16.5-22.7) | 13.5 (11.1-16.4) |
| **Educational level** |  |  |
| No college/university | 17.3 (15.3-19.5) | 12.8 (11.1-14.8) |
| College/university | 14.2 (11.3-17.6) | 10.5 (8.0-13.5) |
| **Duration of diabetes** |  |  |
| <1 years | 24.1 (14.8-36.7) | 15.8 (8.4-27.7) |
| 1-4 years | 18.2 (14.4-22.7) | 10.2 (7.4-13.9) |
| 5-9 years | 14.1 (10.7-18.3) | 8.8 (6.2-12.4) |
| ≥ 10 years | 15.5 (13.3-18.0) | 14.3 (12.2-16.8) |
| **HbA_1c_ categories** |  |  |
| ≤7.0 % (≤53 mmol/mol) | 16.7 (14.7-19.0) | 12.4 (10.6-14.4) |
| 7.1-7.5 % (54-58 mmol/mol) | 15.6 (11.5-20.8) | 11.8 (8.2-16.6) |
| 7.6-8.0 % (59-64 mmol/mol) | 13.7 (9.1-20.1) | 7.7 (4.4-13.4) |
| 8.1-8.9 % (65-74 mmol/mol) | 17.8 (11.9-25.8) | 13.3 (8.3-20.7) |
| ≥9.0 % (≥75 mmol/mol) | 13.7 (6.7-26.1) | 17.3 (9.3-30.0) |
| **Using insulin now** |  |  |
| Yes | 15.4 (12.1-19.5) | 15.6 (12.3-19.7) |
| No | 16.6 (14.7-18.7) | 11.1 (9.6-13.0) |
| **Microvascular complications** |  |  |
| Diabetes eye- problems |  |  |
| No | 10.2 (8.7-11.9) | 11.7 (10.2-13.3) |
| Yes | 16.7 (11.3-23.8) | 21.4 (13.9-31.5) |
| Diabetes foot-ulcers |  |  |
| No | 11.7 (10.2-13.3) | 10.2 (8.7-11.9) |
| Yes | 21.4 (13.9-31.5) | 18.9 (11.3-23.8) |
| Any microvascular complication# |  |  |
| No | 16.0 (14.2-17.9) | 11.5 (10.0-13.1) |
| Yes | 20.1 (14.2-17.9) | 17.8 (13.2-23.6) |
| **Macrovascular complications** |  |  |
| Acute myocardial infarction |  |  |
| No | 16.9 (15.0-18.9) | 11.8 (10.2-13.6) |
| Yes | 12.0 (8.4-16.9) | 14.0 (10.1-19.1) |
| Cerebral stroke |  |  |
| No | 16.5 (14.7-18.5) | 11.5 (10.0-13.3) |
| Yes | 14.7 (9.6-21.9) | 18.9 (13.1-26.5) |
| Angina Pectoris |  |  |
| No | 16.9 (15.1-18.9) | 11.7 (10.1-13.4) |
| Yes | 10.1 (6.3-15.8) | 14.9 (10.1-13.4) |
| Any macrovascular complication≠ |  |  |
| No | 17.4 (15.5-19.5) | 11.5 (9.9-13.3) |
| Yes | 13.1 (10.1-16.8) | 14.9 (11.5-18.4) |

# diabetes eye problem and/or history of diabetes foot ulcer.

≠ acute myocardial infarction and/or cerebral stroke and/or angina pectoris.
